# *Aspergillus flavus* grown in peptone as the carbon source exhibits spore density- and peptone concentration-dependent aflatoxin biosynthesis

**DOI:** 10.1186/1471-2180-12-106

**Published:** 2012-06-13

**Authors:** Shijuan Yan, Yating Liang, Jindan Zhang, Chun-Ming Liu

**Affiliations:** 1 Practaculture College, Gansu Agricultural University, Lanzhou, 730070, China; 2 Key Laboratory of Plant Molecular Physiology, Institute of Botany, Chinese Academy of Sciences, 20 Nanxincun, Fragrant Hill, Beijing,, 100093, China; 3 School of Biology and Basic Medical Sciences, Soochow University, Suzhou, 215000, China

**Keywords:** *A. flavus*, *A. parasiticus*, *A. nomius*, Peptone, Aflatoxin biosynthesis, Density effect

## Abstract

**Background:**

Aflatoxins (AFs) are highly carcinogenic compounds produced by *Aspergillus* species in seeds with high lipid and protein contents. It has been known for over 30 years that peptone is not conducive for AF productions, although reasons for this remain unknown.

**Results:**

In this study, we showed that when *Aspergillus flavus* was grown in peptone-containing media, higher initial spore densities inhibited AF biosynthesis, but promoted mycelial growth; while in glucose-containing media, more AFs were produced when initial spore densities were increased. This phenomenon was also observed in other AF-producing strains including *A. parasiticus* and *A. nomius*. Higher peptone concentrations led to inhibited AF production, even in culture with a low spore density. High peptone concentrations did however promote mycelial growth. Spent medium experiments showed that the inhibited AF production in peptone media was regulated in a cell-autonomous manner. mRNA expression analyses showed that both regulatory and AF biosynthesis genes were repressed in mycelia cultured with high initial spore densities. Metabolomic studies revealed that, in addition to inhibited AF biosynthesis, mycelia grown in peptone media with a high initial spore density showed suppressed fatty acid biosynthesis, reduced tricarboxylic acid (TCA) cycle intermediates, and increased pentose phosphate pathway products. Additions of TCA cycle intermediates had no effect on AF biosynthesis, suggesting the inhibited AF biosynthesis was not caused by depleted TCA cycle intermediates.

**Conclusions:**

We here demonstrate that *Aspergillus* species grown in media with peptone as the sole carbon source are able to sense their own population densities and peptone concentrations to switch between rapid growth and AF production. This switching ability may offer *Aspergillus* species a competition advantage in natural ecosystems, producing AFs only when self-population is low and food is scarce.

## Background

Aflatoxins (AFs) are a group of polyketide metabolites produced by several toxigenic species of *Aspergillus* such as *A. flavus* and *A. parasiticus* after infections of seeds with high protein and lipid contents, e.g. peanut, corn and walnut [1-3]. AFs are toxic and carcinogenic, posing serious threats to both animal and human health [4]. Extensive studies carried out in *A. flavus* and *A. parasiticus* lead to the identification of a 70 kb DNA cluster consisting two specific transcriptional regulators (*aflR* and *aflS*), and 26 co-regulated downstream metabolic genes in the AF biosynthetic pathway [5-8]. Expressions of *aflR* and *aflS* are further regulated by global regulators such as the CreA transcription factor and the VelB/VeA/LaeA complex, and possibly by a cell surface-localized G-protein coupled receptor complex [2,9,10].

Various nutritional and environmental factors including carbon sources [11], nitrate [12], light [13], temperature [14,15], pH [14,16], and oxygen availability [17-19] affect AF productions and expressions of AF biosynthesis-related genes [9,20,21]. It has been known for a long time that sugars and related carbohydrates support both fungal growth and AF production. However, peptone, a mixture of protein degradation products, is a preferred carbon source for fungal growth, but not for AF production [11,22-25]. Many studies have been carried out to elucidate how various carbon sources affect AF biosynthesis. Transition from peptone mineral salts (PMS) medium to glucose mineral salts (GMS) medium leads to AF biosynthesis, a process requiring de novo transcription and translation [24]. Comparisons of a large collection of carbon sources reveal that sugars that are normally oxidized through the hexose monophosphate or glycolytic pathway such as glucose, raffinose and mannose are efficient carbon sources for AF productions [23], while lactose and most amino acids excluding aspartate are considered to be unsuitable carbon sources for AF production [11,26]. AFs are usually produced in parallel with fatty acid biosynthesis following the rapid growth and sugar utilization phase, as common precursors such as acetyl-CoA and malonyl-CoA derived from glucose catabolism are utilized in both pathways [18]. As many carbohydrates are able to induce AF production, Abdollahi and Buchanan (1981) believe that utilization of readily metabolized carbohydrates may result in elevated energy status which in turn induces AF biosynthesis [23]. Wiseman and Buchanan (1987) note that, although mycelia grow well in media with low concentrations of suitable sugars, AFs are produced only when sugar concentrations are higher than 0.1 M, and in which reduced mycelial growth and inhibited TCA cycle activity are observed [27]. Addition of TCA cycle intermediates inhibits AF production, suggesting that glucose may regulate AF productions through inhibition of the TCA cycle [25,26]. Recent studies have revealed cell density-dependent sclerotium formation and AF production in media with glucose and sorbitol as the carbohydrate sources, which is regulated through non-cell autonomous factors [28,29].

In nature, seeds with high protein and lipid content, such as peanut and cotton, are more susceptible to high AF production than starchy seeds like rice and sorghum [1]. It has also been shown in maize that mycelial growth and AF production occur primarily in the embryo and the aleurone layer where mainly storage proteins and lipids are accumulated [30,31]. Removal of oil from ground cotton seeds greatly enhances AF production, suggesting that lipids are not essential for optimal AF biosynthesis [32]. Fatty acids may stimulate or inhibit AF production through the presence of various oxidation-derived oxilipins [33-36]. The influence of protein and peptone on AF biosynthesis remains largely unknown.

In this study we investigated how AF production by *Aspergillus* was influenced when peptone was used as the sole carbon source. Contrary to expectations, we observed spore density- and peptone concentration-dependent AF production with peptone as the sole carbon source. AFs were only produced in the PMS medium when initial spore densities were 10^4^ spores/ml or lower. In contrast, mycelia cultured in the PMS medium with higher initial spore densities or with increased peptone concentrations grew rapidly but without AF production. Spent media experiments showed that no inhibitory factors were released into the culture media. Metabolomic analyses revealed that, in addition to inhibited AF biosynthesis, mycelia grown in peptone media with high initial spore densities showed enhanced sugar utilization and repressed lipid biosynthetic metabolism.

## Results

### Spore density-dependent AF production in PMS media

**Figure 1:**
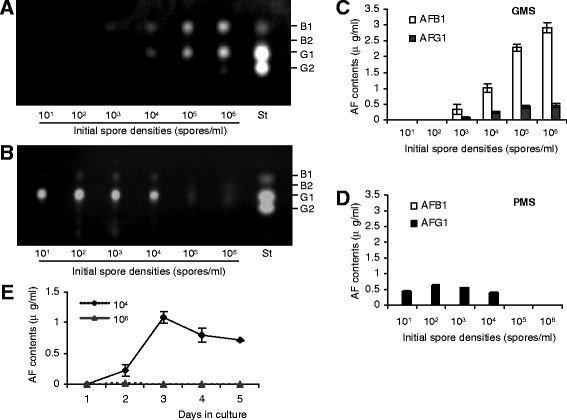
**Spore density-dependent AF productions in*A. flavus*in PMS media.** (**A**, **B**), TLC analyses of AF productions by *A. flavus* A3.2890 cultured in GMS (A) or PMS (B) media for 3 days with initial spore densities of 10^1^, 10^2^, 10^3^, 10^4^, 10^5^ and 10^6^ spores/ml. Ten μl AF extracts were loaded in (A), and 50 μl in (B). St: AF standards. (**C**, **D**) HPLC analyses of AFs produced by *A. flavus* A3.2890 cultured in GMS (C) or PMS (D) media for 3 days, with the initial spore densities of 10^1^, 10^2^, 10^3^, 10^4^, 10^5^ and 10^6^ spores/ml. Note in GMS media both AFB1 and AFG1 were produced, while in PMS media mainly AFG1 was produced. (**E**) The time course of AFG1 productions in PMS media during 5-day cultures, with initial spore densities of 10^6^ (dotted line) or 10^4^ (solid line) spores/ml. All results were the mean ± SD of 3 measurements from mixed three independent samples.

PMS has long been considered to be a non-conducive medium for AF production in both *A. flavus* and *A. parasiticus*[23-25]. To investigate the mechanism underlying peptone’s influence on AF biosynthesis, the well-studied *A. flavus* A3.2890 [37-39] from the China General Microbiological Culture Collection Center (CGMCC) was used to conduct our experiments. It was indeed the case that *A. flavus* did not produce AFs when cultured at the commonly employed initial spore density of 10^5^ or 10^6^ spores/ml. However, when various spore densities of *A. flavus* were tested to initiate cultures, a density-dependent AF production was observed. When the initial spore density was gradually decreased, increasing amounts of AFs were detected in media after 3-day culture, as shown by thin-layer chromatography (TLC) and high pressure liquid chromatography (HPLC) analyses (Figure 1B & D). At 10^1^ spores/ml, the amount of AFs produced was significantly lower, comparable to that of the 10^4^ spores/ml culture. The maximal AF production was observed in the PMS medium inoculated with 10^2^ spores/ml. This differs from GMS cultures, where increasing amounts of AFs were produced when initial spore densities were increased from 10^1^ to 10^6^ spores/ml (Figure 1A & C). We also observed that in GMS media, AFB1 was the major toxin (Figure 1C), while in PMS media, AFG1 was the primary toxin produced (Figure 1D). These data suggest that AF biosynthesis is regulated differentially in these two media.

Since most *A. flavus* strains produce only AFB1 [40-42], we examined if the A3.2890 strain used was indeed *A. flavus*. By using the protocol developed by Henry *et al* (2000) [43], fragments of the internal transcribed spacer (ITS) region of *rRNAβ*-Tubulin and Calmodulin genes from the *A. flavus* A3.2890 strain were amplified and sequenced, and then compared with corresponding sequences in the Genbank, and confirmed that A3.2890 is indeed *A. flavus* (see Additional files 1,2,3 and 4). It is very likely that the strain we used belongs to the type IV *A. flavus*, which produces both AFBs and AFGs, as reported recently [44].

### The time course of AF production

To assess the production and possible degradation of AFs during the cultural period with various initial spore densities, we examined AFG1 contents in the PMS medium during a five-day culture period, with 10^6^ or 10^4^ spores/ml. We observed that, in the culture initiated with 10^4^ spores/ml, a significant amount of AFG1 was detected on the day two, reached the maximum level on the day three, and subsequently decreased gradually. In contrast, almost no AFs were detected in the culture initiated with 10^6^ spores/ml during the entire five-day culture period (Figure 1E). It has been shown previously that peptone from different suppliers may induce different enzyme activities in *Candida albicans*[45]. The peptone initially used in this study was purchased from Beijing Aoboxing Biotech. To ensure the result observed is a general phenomenon, peptone from Sigma and Shuangxuan Microbe Culture Medium Products Factory was tested, and same results were observed (see Additional file 5).

To examine if cultures with high initial spore densities lead to a similar AF accumulation in mycelia, we used the TLC method to analyze AF contents in mycelia cultured for three days in either PMS or GMS media, with 10^4^ or 10^6^ spores/ml. The results showed greatly reduced AF content in mycelia in culture initiated with 10^6^ spores/ml, similar to the AF content of the media. In contrast, increased AF production was observed in mycelia cultured in GMS media with 10^6^ spores/ml, as compared to that with 10^4^ spores/ml (see Additional file 6).

### High initial spore density in PMS media led to rapid mycelial growth

**Figure 2:**
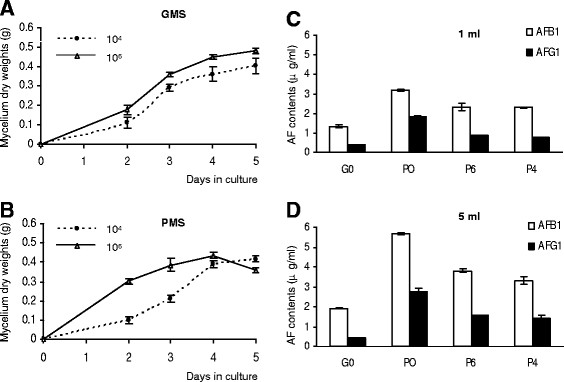
**Mycelial growth and AF production of*A. flavus*cultured with different initial spore densities.** (**A**, **B**) Mycelial growth curves of *A. flavus* A3.2890 in 50 ml GMS (**A**) or PMS (**B**) media initiated with 10^4^ (dotted line) or 10^6^ spores/ml (solid line). The mycelium dry weights were measured during a period of 5 days. (**C**, **D**) Effects of PMS spent media on AF productions. (**C**) One ml fresh GMS (G0) or PMS (P0) media, or spent media (P4 and P6) were added to GMS media inoculated with 10^6^ spores/ml. (**D**) Five ml fresh GMS (G0) or PMS (P0), or spent media (P4 and P6) were added to GMS media inoculated with 10^6^ spores/ml. AF contents were measured after cultured at 28°C for 3 days. The spent media were prepared from 3-day PMS cultures with the initial spore densities of 10^4^ (P4) or 10^6^ (P6) spores/ml. All data were the mean ± SD of 3 HPLC measurements from mixed three independent samples.

To exclude the possibility that the reduced AF production in PMS media initiated with high initial spore densities was caused by inhibited fungal growth, mycelium dry weights were determined during a five-day culture period. *A. flavus* cultured in GMS media with an initial density of 10^4^ or 10^6^ spores/ml showed a similar growth curve, with a continuous increase in dry weight during the five-day incubation. Higher initial spore density led to slightly faster mycelial growth, and an increased mycelium dry weight (Figure 2A). *A. flavus* cultured with 10^4^ spores/ml in PMS media showed a similar growth curve to that in GMS media with the same spore density (Figure 2B). However, a much sharper exponential growth phase was observed in the first two days in PMS culture initiated with 10^6^ spores/ml (Figure 2B). The mycelium dry weight reached the maximum level on the 4^th^ day and decreased significantly afterwards, suggesting no inhibition of growth in the high density PMS culture. Instead, *A. flavus* cultured in PMS media with a high initial spore density grew faster and degenerated earlier (Figure 2B).

### No inhibitory factor was released from the high density culture into the media

We examined whether inhibitory factors were released into the media by *A. flavus* grown in PMS media with high initial spore densities. The experiment was performed by adding filter-sterilized spent media collected from 3-day cultures with 10^4^ or 10^6^ spores/ml to fresh GMS media inoculated with 10^6^ spores/ml. Filter-sterilized fresh PMS or GMS media were used as controls. The addition of 1 ml fresh PMS medium (P0) to GMS cultures enhanced production of both AFB1 and AFG1, as compared to the addition of fresh GMS medium (G0) (Figure 2C), which is in agreement with a previous report [46]. As showed in Figure 2C, addition of 1 ml spent media from both high (without AF production) and low (with AF production) density cultures to the GMS culture promoted AF production. No significant difference in AF production was observed in the high density culture. The experiment was extended further to add 5 ml spent media from high (P6) and low (P4) density cultures. If inhibiting factors were present in the spent media, we would expect to see reduced AF productions when compared to addition of 1 ml spent media. However, we observed that more AFs were produced in both P4 and P6 cultures, and no significant difference was observed between P4 and P6 samples (Figure 2D). Lower levels of AFs were produced in cultures with spent PMS media than those with fresh PMS media (Figure 2C & D), which could be explained by nutrient consumption during the three-day incubations. These data together show that there seems to be no inhibitory factor released from the high density culture to the media.

### Increased peptone concentrations inhibited AF production

**Figure 3:**
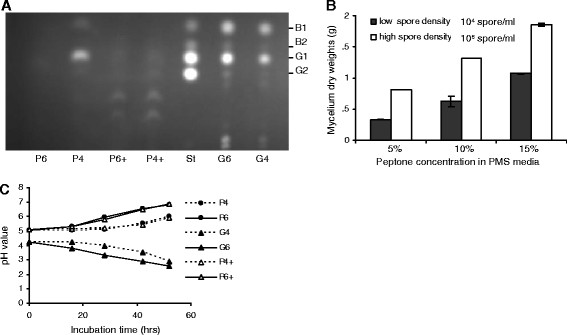
***A. flavus*grown in PMS and GMS media responded differently to the initial spore densities.** (**A**) Higher concentrations of peptone inhibited AF productions in *A. flavus* A3.2890. P4, PMS media with the initial spore density of 10^4^ spores/ml; P6, PMS media with the initial spore density of 10^6^ spores/ml; G4, cultured in GMS media with the initial spore density of 10^4^ spores/ml; G6, cultured in GMS media with the initial spore density of 10^6^ spores/ml; P4+, PMS media with 15% peptone, cultured with the initial spore density of 10^4^ spores/ml; P6+, PMS media with 15% peptone, cultured with the initial spore density of 10^6^ spores/ml, St, AF standard. (**B**) Higher concentrations of peptone promoted mycelial growths. The total mycelium dry weights were measured after a 3-day culture, with initial spore densities of 10^4^ or 10^6^ spores/ml. (**C**) No direct correlations between AF productions and pH changes. In GMS media the pH was gradually decreased during the 55-hr culture, where a higher initial spore density led to faster acidification of the medium. In PMS media the pH was increased during culture, where a higher initial spore density led to rapid alkalization of the medium. Note that increased peptone concentrations did not cause a significant change in the pH of PMS media (P6 and P6+).

To examine if the lack of AF production in PMS media with high initial spore densities is caused by rapid mycelial growth, and consequent depletion of nutrients, the peptone concentration in media from the original 5% was increased to 15% to see if AF production could be restored. We observed, conversely, that mycelia cultured with increased peptone concentration showed greatly reduced AF production, regardless of initial spore densities (10^4^ or 10^6^ spores/ml) (Figure 3A, P4+ and P6+). We then examined the mycelial growth in media with 5%, 10% and 15% peptone, and observed increased mycelium dry weights when the peptone concentrations were increased (Figure 3B), suggesting that high concentrations of peptone promoted mycelial growth and at the same time inhibited AF biosynthesis. For each of the peptone concentrations, it was observed that cultures with higher initial spore densities showed an increase in mycelial growth. Taken together, these studies revealed that high concentrations of peptone promoted mycelial growths but inhibited AF production, suggesting that *A. flavus* grown in the peptone medium is able to sense the peptone concentrations and is able to shift between fast growth and AF production.

It has been reported previously that carbon sources affect the pH of culture media [14,16]. If AF production in media correlates with the pH changes was examined during incubation. We found that, as reported by Buchanan and Lewis (1984) [25], the pH of cultures in GMS media was decreased (Figure 3C, G4 and G6), while pH of cultures in PMS media was increased during the 55-hr cultures (Figure 3C, P4, and P6). Higher initial spore density led to faster acidification or alkalization of the GMS and PMS media during the cultures, respectively (Figure 3C). Interestingly, we observed that when the peptone concentration was increased to 15%, the pH of the media increased in the same way as the 5% peptone media (Figure 3C, P4+ and P6+). AF production was inhibited in the medium with 15% peptone, while AF production was active in the medium with 5% peptone, which suggests no direct connection between AF production and pH changes in our incubation system.

### High initial spore densities in PMS media repressed the expression of AF biosynthesis-related genes

**Figure 4:**
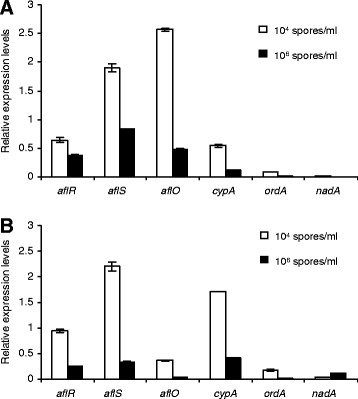
**High initial spore densities repressed the expressions of AF biosynthesis genes in*A. flavus.*** qRT-PCR was used to analyze expressions of AF production regulators (*aflR* and *aflS*) and AF biosynthesis genes (*aflO*, *cypA*, *ordA* and *nadA*) by *A. flavus* A3.2890 cultured in PMS media with 10^4^ or 10^6^ spores/ml for 2 (**A**) or 3 days (**B**). The relative expressions were quantified by the expression level of the *β-Tubulin* gene. Note the expression of *nadA* was not repressed in the high initial spore density culture.

To further study how initial spore densities affect AF production in *A. flavus*, expression of AF biosynthesis-related genes was examined by quantitative reverse transcription PCR (qRT-PCR) in mycelia initiated with 10^4^ or 10^6^ spores/ml for two days. We observed that the expression levels of two transcriptional regulators (*alfR* and *alfS*), and three AF biosynthesis genes (*aflO, cypA and ordA*) from the AF biosynthesis gene cluster were substantially lower in mycelia initiated with 10^6^ spores/ml, as compared to those initiated with 10^4^ spores/ml (Figure 4A). The differences were even more pronounced on the day three (Figure 4B), suggesting transcriptional activation of AF biosynthesis in cultures initiated with the low spore density. We noted that *nad*A, which is involved in the conversion of AFG1 [47], showed increased expression in the culture initiated with 10^6^ spores/ml, compared to those initiated with 10^4^ spores/ml on the day three (Figure 4B).

### The density effect was present in most *Aspergillus* strains tested

**Figure 5:**
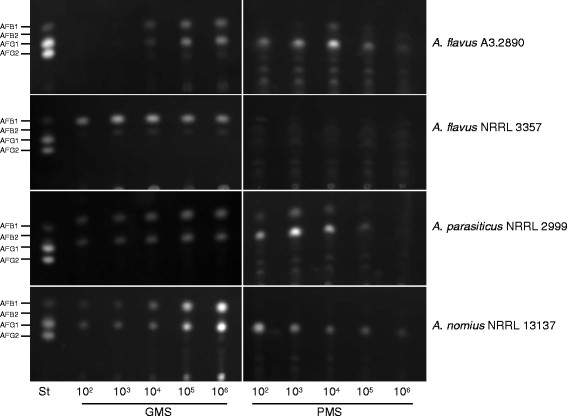
**The density effect is present in all*Aspergillus*strains tested except*A. flavus*NRRL 3357.** Strains of *A. flavus* NRRL 3357, *A. parasiticus* NRRL 2999 and *A. nomius* NRRL 13137 were tested for their density effects. Freshly prepared spores at the densities of 10^2^ to 10^6^ were cultured in GMS and PMS media and AF contents were analyzed by TLC after 3 days. Note no AF was produced in PMS media by *A. flavus* NRRL 3357. St: AF standards.

To elucidate if the density effect is a general phenomenon in AF-producing strains, we obtained *A. flavus* NRRL 3357, *A. parasiticus* NRRL 2999 and *A. nomius* NRRL 13137 from the Agricultural Research Service (ARS) culture collection in United States Department of Agriculture (USDA), and performed experiments in parallel with *A. flavus* A3.2890. Fresh spore suspensions were prepared in the same way as for *A. flavus* A3.2890, and inoculated in PMS or GMS liquid media with initial spore densities from 10^2^ spores/ml to 10^6^ spores/ml. After three-day cultures, AFs were extracted from media and analyzed by TLC. As shown in Figure 5, in GMS media, all strains showed increased AF productions when initial spore densities were increased from 10^2^ to 10^6^ spores/ml, excluding *A. flavus* NRRL 3357. As reported previously, only AFB1 and AFB2 were produced by *A. flavus* NRRL 3357 [48], while for all other strains AFB1 and AFG1 were the major AFs produced.

In PMS media, similar to what was showed above in *A. flavus* A3.2890, we observed that high initial spore densities inhibited AF biosynthesis in *A. parasiticus* NRRL 2999 and *A. nomius* NRRL 13137, especially when initial spore densities were 10^5^ spores/ml or higher (Figure 5). However, no AF biosynthesis was observed in *A. flavus* NRRL 3357 in PMS media, no matter the initial spore density. It seems somehow the *A. flavus* NRRL 3357 strain has lost the density sensing machinery in evolution.

### Mycelia grown in PMS media with high initial spore densities showed reduced TCA cycle intermediates and fatty acid accumulations, but enhanced PP pathway products

**Figure 6:**
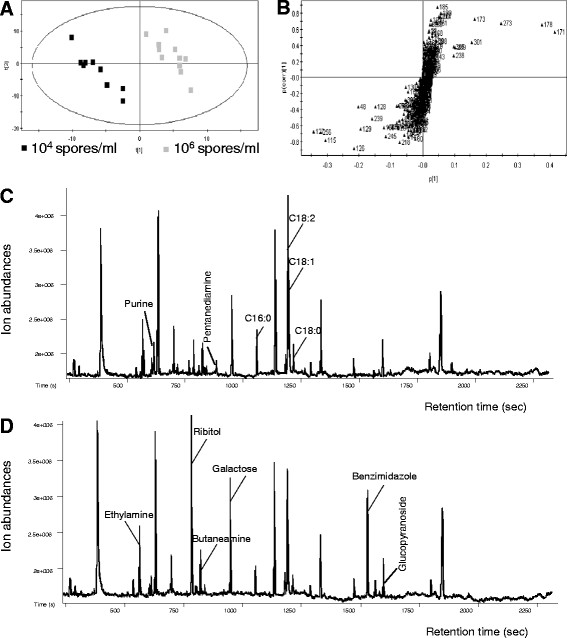
**Metabolites with different contents in cultures initiated with high or low spore densities.** (**A**) A PLS scores plot, performed using SIMCA-P V11.0, for metabolites extracted from mycelia cultured for 2, 3, 4 and 5 days in PMS media with initial spore densities of 10^4^ (black) and 10^6^ (gray) spores/ml, with 3 replicates in each treatment. (**B**) Scatter loading plots obtained from PLS analyses of the entire GC-Tof-MS dataset. (**C** and **D**) Total ion chromatographies of metabolites extracted from mycelia of *A. flavus* grown in PMS media for 3 days with the initial spore densities of 10^4^ (C) and 10^6^ spores/ml (D). Metabolites with significant differences in quantity between (C) and (D) are labeled.

To determine metabolic differences in *A. flavus* grown in PMS media with high or low initial spore densities, metabolites in mycelia cultured for 2, 3, 4 and 5 days were analyzed by gas chromatography time-of-flight mass spectrometry (GC-Tof-MS) using methods described previously [49,50]. Multi-variate analyses showed that mycelia inoculated with 10^4^ spores/ml clustered separately from mycelia inoculated with 10^6^ spores/ml, suggesting evident metabolic differences between these two cultures (Figure 6A & B). Striking differences in levels were observed in 24 metabolites on the 3^rd^ day (Figure 6C & D, and Table 1). In PMS cultures initiated with 10^6^ spores/ml, a condition without AF production, the level of three TCA cycle intermediates, namely malic acid, fumaric acid and succinic acid, accumulated significantly less than those in cultures initiated with 10^4^ spores/ml This suggests that the TCA cycle was more active in the high density culture. Similarly, levels of four fatty acids, palmitic acid, stearic acid, oleic acid and linoleic acid, were reduced in cultures initiated with the high spore density (Table 1), indicating that fatty acid biosynthesis was generally inhibited in the high density culture. In contrast, many sugar metabolites including ribitol, glucopyranoside, gluconolactone-6-P, glycerol, butanediamine, ethylamine and galactose, were accumulated more in the high density cultures (Table 1), suggesting that the PP pathway was active. In addition, nucleotides and compounds involved in amino acid metabolism were less abundant in cultures initiated with the high spore density (Table 1), which may be the consequence of the rapid mycelial growth.

**Table 1:** Metabolites differ between high and low spore density cultures

**Groups**	**Metabolites**	**R. T.**	**Relative contents**	**Relative contents**	**10**^**4**^**/10**^**6**^
		**(sec)**	**in 10**^**4**^spores/ml*	**in 10**^**6**^spores/ml*	**ratio**
**TCA cycle intermediates**	Malic acid	587.5	0.0187 ± 0.0001	0.0098 ± 0.0027	1.91
	Fumaric acid	1349.0	0.0869 ± 0.0090	0.0509 ± 0.0043	1.71
	Succinic acid	269.8	0.0103 ± 0.0020	0.0043 ± 0.0016	2.4
**Amino acids**	Glycine	417.0	0.0066 ± 0.0028	0.0034 ± 0.0003	1.94
	Phenylalanine	713.7	0.1649 ± 0.0330	0.0084 ± 0.0007	19.63
	Proline	410.0	0.0552 ± 0.0179	0.0099 ± 0.0009	5.58
	Valine	331.5	0.0191 ± 0.00028	0.0088 ± 0.0006	2.17
	Tyrosine	964.1	0.0430 ± 0.0090	0.0242 ± 0.0027	1.78
	Isoleucine	404.0	0.0059 ± 0.0017	0.0042 ± 0.0003	1.4
**Fatty acids**	Palmitic acid	1054.5	0.3447 ± 0.037	0.2640 ± 0.011	1.31
	Stearic acid	1212.4	0.2218 ± 0.027	0.1402 ± 0.0133	1.58
	Oleic acid	1193.0	0.0833 ± 0.0024	0.0744 ± 0.0033	1.12
	Linoleic acid	1188.8	0.8959 ± 0.0671	0.6315 ± 0.0554	1.42
**Nucleotides**	Pyrimidine	449.0	0.0154 ± 0.0044	0.0039 ± 0.0004	3.95
	Purine	630.8	2.1901 ± 0.3141	1.3194 ± 0.0221	1.66
**AA metabolism**	Pentanediamine	881.1	0.1703 ± 0.0143	0.0162 ± 0.0011	10.51
	Amine	369.0	0.0734 ± 0.0261	0.0328 ± 0.0036	2.24
**Sugar metabolism**	Ribitol	784.2	0.6276 ± 0.1768	2.6039 ± 0.1502	0.24
	Glucopyranoside	1561.8	0.1130 ± 0.0198	0.3344 ± 0.0354	0.34
	Gluconolactone-6-P	821.6	0.5679 ± 0.0839	0.7094 ± 0.0181	0.80
	Glycerol	986.0	0.0073 ± 0.0015	0.0103 ± 0.0009	0.71
	Butanediamine	801.1	0.0656 ± 0.0086	0.1224 ± 0.0051	0.54
	Ehylamine	563.0	0.2082 ± 0.0320	0.2436 ± 0.0013	0.85
	Galactose	948.1	1.6122 ± 0.4037	1.9547 ± 0.3306	0.82

*Values represent metabolites abundances (mean ± SD) of 3 replicates from mixed 3 independent samples, as measured by GC-Tof-MS in 3-day mycelia (normalized by C17:0 fatty acid). R.T.: retention time; TCA: tricarboxylic acid; AA: amino acid.

### Addition of TCA cycle intermediates did not affect AF biosynthesis

**Figure 7:**
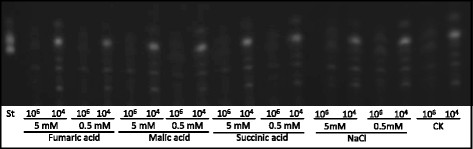
**Additions of TCA cycle intermediates can not restore the AF biosynthesis in high initial spore density cultures.** In *A. flavus* A3.2890 mycelia grown in PMS media initiated with 10^4^ and 10^6^ spores/ml, 0.5 mM or 5 mM TCA cycle intermediates, fumaric acid, malic acid and succinic acid, were added at the beginning of the culture. AFs were extracted from media and analyzed by TLC after 3-day cultivations.

To test if reduced TCA cycle intermediates in mycelia are the primary cause of reduced AF biosynthesis in the high initial spore density culture, malic acid, fumaric acid and succinic acid were added to the PMS medium at the concentrations of 0.5 mM or 5 mM, and 0.5 or 5 mM NaCl was added to the culture as a control, and then performed liquid incubation with the final spore densities of 10^4^ or 10^6^ per ml using freshly prepared *A. flavus* A3.2890 spore suspensions. TLC analyses were performed for AFs extracted from the media. We observed that none of these treatments had any significant effect on AF production. No AF production was observed in any of the high initial spore density cultures (Figure 7). These results suggest that the inhibited AF biosynthesis in high initial spore density cultures was unlikely caused by reduced levels of TCA cycle intermediates.

## Discussion

As a group of highly toxic natural compounds, AFs in nature are produced mainly in seeds with high lipid and protein content [1,3]. Previous reports show that peptone is not a suitable carbon source for AF production [23-25]. Our present study demonstrates that peptone was in fact conducive for AF production, as long as the initial spore density of *A. flavus* was reduced. Mycelia grown in peptone media responded not only to the initial spore density, but also to peptone concentration. Higher initial spore density and higher concentration of peptone inhibited AF biosynthesis. We also showed that no AF biosynthesis inhibitor was released into the media in the culture with the higher initial spore density. qRT-PCR analyses revealed that culture with a high initial spore density repressed expression of both the transcriptional regulators and the biosynthesis genes in the AF pathway gene cluster. Metabolomic studies showed that, in high density cultures, the TCA cycle and PP pathway were active, while the fatty acid biosynthesis pathway was repressed.

### Spore density- and peptone concentration-dependent AF biosynthesis in PMS media

In nature, many organisms, especially fungal species, are able to produce compounds to suppress the growth of other organisms in their neighborhood [51]. Regulated production of these compounds is expected to have physiological and ecological advantage for these organisms. It has been shown previously that lower glucose content supports fungal growth but not AF accumulation, suggesting that the first priority of the fungus is growth when food availability is low [27]. In our study we observed that mycelia grown in peptone media showed spore density- and peptone concentration-dependent AF production in *A. flavus*. High initial spore density or high peptone concentration promoted rapid mycelial growth without AF biosynthesis, which may allow the fungus to prioritize propagation when the competition pressure is low, and when sufficient food is available. In contrast, active AF productions were observed in cultures initiated with lower spore densities and lower concentrations of peptone. Additional comparative studies using several AF-producing strains including *A. flavusA. parasiticus* and *A. nomius* from the USDA ARS culture collection showed that the density-dependent AF biosynthesis in PMS media was present in all strains tested except *A. flavus* NRRL 3357. This particular strain did not produce any AFs in PMS media, as reported previously [52]. Furthermore, a positive correlation between AF production and initial spore density in GMS media was also not observed for this strain, implicating a different regulation mechanism is evolved. In natural ecosystems where the self population density is low and food is scarce, AF production may confer competitive advantages, through inhibition of the growth of other organisms. It would be interesting to examine if other fungal species also employ this survival strategy.

We showed that no soluble AF biosynthesis inhibitor was released from the high spore density culture to media by using spent medium experiments, suggesting that *A. flavus* A3.2890 is somehow able to sense the population density and adjust their growth and AF production through cell-autonomous machinery. Unlike *Candidia albicans* and *Dictyostelium,* where density factors are diffusible to media [53-55], we hypothesize that *A. flavus* may use a cell surface component to perceive such cultural density and nutrient signals. The possible role of G protein-mediated signaling [56] in this process is worth exploring. Alternatively, it has been reported that oxidative stress is a prerequisite for AF production [57]. It is plausible that the rapid growth in PMS media with high initial spore densities may lead to reduced intracellular oxygen availability and subsequently decreased oxidative stress, which could prevent AF production. It will be interesting to examine why this density-sensing machinery is active only when peptone, not glucose, is used as the carbon source.

### High initial spore densities repressed expression of AF biosynthesis- related genes including *aflS* and *aflR*

Transferring *A. parasiticus* mycelia from PMS to GMS media resulted in AF production, which is inhibited by cycloheximide or actinomycin D treatments, suggesting both *de novo* transcription and translation are required for AF biosynthesis [23,24]. In this study, we observed that high initial spore densities promoted mycelial growth but inhibited AF production, which is similar to the high temperature cultures in GMS media where no AFs are produced [58]. High temperature culture (37°C) specifically represses the expressions of AF biosynthesis genes without affecting expression of the transcriptional regulators *aflR* and *aflS* in the AF pathway gene cluster [20,59,60]. However, we found that high initial density cultures inhibited the expression of both the transcriptional regulators (*aflR* and *aflS*) and downstream AF biosynthesis genes simultaneously, suggesting a different manner of regulation. Further study is needed to elucidate if the density-dependent AF biosynthesis is regulated through antagonistic signaling pathways that coordinate vegetative growth, conidiation and AF production [2].

### Cultures with high initial spore densities in PMS media trigger a metabolic shift from AF production to sugar metabolism

Although primary and secondary metabolism share common transcriptional and translational machinery, secondary metabolism often commences during idiophase, when normal growth and development have ceased [61]. The present study revealed a novel low cell density-dependent metabolic switch toward AF production in *A. flavus*. We observed that the AF and lipid biosynthesis were active in mycelia initiated with a low spore density in the PMS medium. In contrast, the TCA cycle was inhibited, as shown by the accumulation of TCA cycle intermediates in low spore density cultures, which is in agreement with previous results showing that the TCA cycle is repressed during active AF biosynthesis, to allow a greater acetyl-CoA shunt toward AF biosynthesis [26]. By adding three TCA cycle intermediates to cultures, we showed that increased TCA cycle intermediates did not restore AF biosynthesis in the high initial spore density culture, nor did additional TCA cycle intermediates promote AF biosynthesis in the low spore density culture, suggesting that the spore density-regulated AF biosynthesis in the PMS medium is not likely influenced by TCA cycling directly. The enhanced TCA cycling might be the consequences of inhibited AF biosynthesis in the high spore density culture.

Since AF production shares a subset of biosynthetic steps with fatty acid metabolism, accumulation of AFs and lipids often occur in parallel [18,62]. This parallel biosynthesis trend was observed in our Metabolomic studies. All four fatty acids detected, palmitic acid, stearic acid, oleic acid and linoleic acid, were accumulated in the low spore density culture, together with AF biosynthesis. The density-dependent metabolic switch from active TCA cycling in high initial spore density cultures to active AF biosynthesis in low initial spore density cultures may represent a shift in metabolic priority that allows *A. flavus* to produce AFs in the protein-rich environment only when its own population density is low.

## Conclusions

Our studies demonstrate that *A. flavus* grown in media with peptone as the carbon source is able to detect its own population density and nutrient availability, and is able to switch between fast growth and AF production. High initial spore density or high peptone concentration led to rapid mycelial growth and inhibited AF production, while low initial spore density or low peptone concentration promoted AF biosynthesis. Inhibited AF biosynthesis in the high initial spore density culture was accompanied by active TCA cycling and rapid mycelial growth. Supplements of TCA cycle intermediates did not restore AF biosynthesis, suggesting the inhibited AF biosynthesis was not caused by depletion of TCA intermediates. Our spent medium experiments showed that the density-sensing factor regulates AF biosynthesis in a cell-autonomous manner. Expression analyses showed that the density factor acts at the transcriptional level to regulate the expressions of both *aflR* and *aflS* transcription regulators and downstream AF biosynthesis genes. Interestingly, Most *Aspergillus* strains including *A. parasiticus* and *A. nomius* tested were shown to be density-dependent AF biosynthesis in PMS media. Only *A. flavus* NRRL 3357 did not exhibit the density-dependent AF biosynthesis, suggesting different regulation machinery is evolved in this strain. We believe that a cell density- or peptone availability-dependent metabolic switch may provide *A. flavus* with a competitive advantage in the natural ecosystem. Whether or not the perception of population density and peptone availability are regulated through the same signaling pathway will require further study.

## Methods

### Fungal strain and growth conditions

The primary strain used in this study, *A. flavus* A3.2890, was obtained from CGMCC, located in the Institute of Microbiology, Chinese Academy of Sciences. *A. flavus* NRRL 3357, *A. parasiticus* NRRL 2999 and *A. nomius* NRRL 13137 strains were obtained from the ARS culture collection in USDA. The GMS medium was prepared as previously described [63], which contains 50 g/L glucose, 3 g/L (NH4)_2_SO4, 2 g/L MgSO4, 10 g/L KH_2_PO4, and 1 ml/L trace element mixture. The pH was adjusted to 4.5 before autoclaving. The PMS medium was identical to GMS except the glucose was replaced by 5% peptone, and pH was adjusted to 5.2, as described previously [24]. All cultures were prepared by following Park's protocol [64] with minor modifications. Sixty μl of *A. flavus* spore suspensions stored at −80°C in glycerol was pre-cultured on potato-dextrose agar plates at 37°C for 4 days. Mature spores on the surface were harvested and re-suspended in sterile distilled water containing 0.05% Tween 20 (Sigma, St. Louis, USA), diluted to a series of spore densities after counting with a haemacytometer. Five ml of spore suspensions of desired density were added to 45 ml PMS or GMS liquid media, cultured on a shaker (180 rpm) at 28°C in the dark.. The pH of the culture media was measured at different time points following inoculation, during a 55-hr culture period. The three brands of peptone used in this study were purchased from Sigma (Cat. No. P6463, St. Louis, USA), Beijing Aoboxing Biotech (Cat. No. 01–001, Beijing, China) and Beijing Shuangxuan Microbe Culture Medium Products Factory (Cat. No. 02-31A, Beijing, China). TCA cycle intermediates, fumaric acid (Cat. No. F8509), malic acid (Cat. No. M1210) and succinic acid (Cat. No. S3674), were purchased from Sigma-Aldrich and added to PMS media at the beginning of the culture.

### Determinations of fungal dry weights and AF contents

For the determination of fungal dry weights, mycelia grown in 50 ml media were harvested at different time points (48, 72, 96, 120 hrs after inoculations) by filtration through two layers of filter paper, washed by sterilized water, and then freezer-dried before weighing. The filtrate was sterilized by passing through a 0.22 μm membrane, which was used for spent media experiments and AF quantifications. For extraction of AFs from media, an equal volume of chloroform was added and the mixture was vortexed and extracted ultrasonically for 15 min. After centrifugation for 6 minutes at 11498.6xg, the organic phase (lower phase) was filtered through a 0.22 μm membrane and dried under nitrogen gas flow, and re-dissolved in fixed volumes of chloroform. The extracts were analyzed by TLC as previously described [65], except the developing solvent was changed to CHCl_3_:H_2_O (9:1, v/v). The AF levels were quantified by HPLC (Agilent 1200, Waldbronn, Germany), equipped with a reverse phase C18 column (150 mm in length and 4.6 mm internal diameter, 5 μm particle size; Agilent), eluted by gradient elution, starting with a mixture of 25% methanol, 20% acetonitrile and 55% water for 3 min, then changed to a 38% methanol water solution for 0.1 min, eluted with 38% methanol for 2.9 min, detected by a DAD analyzer at 360 nm. Quantification was performed by calculating the amount of AF in samples from a standard calibration curve. For the detection of AFs from the mycelia, dried mycelia were ground to a powder, then extracted with acetone with solid-to-liquid ratio 1:10 (g/ml) for 30 minutes, the extract was analyzed by TLC as described above.

### Metabolomic analyses **by GC-Tof-MS**

Mycelia harvested from the 2^nd^ to the 5^th^ day with a 24-hr interval were lyophilized and extracted by ultrasonication for 40 min with 1.5 ml mixed solvents including methanol, chloroform and water (5:2:1, v/v/v), in which 100 μl of 1 mg/ml heptadecanoic acid (C17:0, Sigma, St. Louis, USA) was added as an internal standard. After the centrifugation at 11,000 g for 10 min, 1 ml of supernatant was transferred to a tube with 400 μl chloroform and 400 μl water, vortexed for 15 sec, centrifuged at 11498.6xg for 10 min, and then 400 μl chloroform phase was transferred to a new glass vial, and dried under the nitrogen gas flow. The pellet was re-dissolved in 50 μl 20 mg/ml O-methylhydroxylamin hydrochloride (Sigma, Steinheim, Switzerland) in pyridine, vortexed and incubated at 37°C for 120 min. Afterwards, 100 μl N-methyl-N-trimethylsily trifluoroacetamide (Sigma, Steinheim, Switzerland) was added immediately to the mixture, vortexed and incubated at 37°C on a shaker (150 rpm) for 30 min, The silyl-derivatized samples were analyzed by GC-Tof-MS after cooling to the room temperature using an Agilent 6890 gas chromatography coupled to a LECO Pegasus IV GC-Tof-MS (LECO, USA) with the EI ionization. The column used was VF-5 ms (30 m in length; 250 μm internal diameter, 0.25 μm film thickness; Varian, USA). The MS was operated in a scan mode (start after 4 min; mass range: 50 – 700 m/z; 2.88 sec/scan; detector voltage: 1400 V), in which helium was used as the carrier gas (1 ml/min) with a constant flow mode, a split injector (340°C, 1:50 split) and a flame ionization detector (340°C). The samples were subjected to a column temperature of 100°C for 3 min, raised to 150°C at a rate of 10°C/min, then to 250°C at 5°C/min, finally to 360°C at 10°C/min, and held for 15 min at 360°C. Sample components were identified by comparison of retention times and mass spectra with reference compounds, and matching to the NIST mass spectral database. Metabolite peak areas representing the abundance of metabolites were normalized to the internal standard (heptadecanoic acid). Multivariate analysis was performed using SIMCA-P V11.5 (Umetrics, Sweden) [66,67]. All GC-Tof-MS analyses were conducted with three replicate cultures, mixed before extractions, and measured three times to get the average contents.

### Expression analyses using qRT-PCR

Mycelia were harvested, frozen and ground in liquid nitrogen. Total RNAs from the mycelia were extracted using Trizol (Invitrogen, USA), and polyA mRNAs were purified using PolyAT Rack mRNA Isolation System (Promega, Madison, WI) according to the manufacturers’ manual. All cDNAs were synthesized by reverse transcription reaction performed with ReverTra Ace (Toyobo, Japan) at 42°C for 1 h, and then 85°C for 15 min to stop the reaction. qRT-PCRs were performed using SYBR Green I in a Rotor-Gene 3000 Cycler (Corbett Research, Australia) with primers and temperatures as described in Additional file 7.

## Competing interest

The authors declare that they have no competing interests.

## Authors’ contributions

SY performed most of the experiments, and drafted the manuscript. YL carried out the comparative studies for different strains and experiments for TCA cycle intermediates treatments. JZ carried out the qRT-PCR and molecular characterization of the A3.2890 strain used in this study. CML supervised the study, participated in experimental design, and revised the manuscript. All authors read and approved the final manuscript.

## Supplementary Material

Additional file 1 **Alignment of ITS sequence of the*A. flavus*A3.2890 with ITS sequences from 13 different*Aspergillus*species in GenBank.** The Genbank accession numbers for ITS sequences used are *A. flavus*: AF138287.1, *A. parasiticus*: GU953212.1, *A. sojae*: AB008419.1, *A. tamari*: JF901808.1 *A. pseudotamarii:* DQ467986.1*, A. caelatus*: EU645658.1, *A. nomius*: AF027860, *A. bombycis*: AF338740, *A. niger*: JN545800, *A. arachidicola*: HM560045, *A. fumigatus*: JN153038, *A. terreus*: EF568102, and *A. nidulans*: AF138289.1. Click here for file

Additional file 2 **Homology matrix and phylogenetic tree, calculated based on comparison among the ITS sequence of*A. flavus*A3.2890 and sequences from different*Aspergillus*species in GenBank.** Note that within the 529 bp region, the ITS sequence of *A. flavus* A3.2890 showed 99.6% identity with the corresponding sequence from *A. flavus* (with only 2 SNPs in the entire region), followed by those from *A. parasiticus* (98.7%), *A. sojae* (98.5%), *A. tamari* (98.1%), and *A. pseudotamarii* and *A. caelatus* (97.9%). Note that 97.7% sequence identity was observed between the ITS sequence from *A. flavus* A3.2890 and that from *A. arachidicola* that also produces both AFB and AFG (with 15 SNPs in the same region). Click here for file

Additional file 3 **Alignment and homology matrix of the calmodulin sequence of the*A. flavus*A3.2890 with calmodulin sequences from 19 different*Aspergillus*species in GenBank.** Note that the calmodulin sequence from *A. flavus* A3.2890 showed the highest homology with the calmodulin genes from *A. flavus* and *A. kambarensis*, while *A. kambarensis* is known to be synonymous to *A. flavus,* but without AF production (Varga et al., 2011). Click here for file

Additional file 4 **Alignment and homology matrix of the beta-tubulin sequence of the*A. flavus*A3.2890 with beta-tubulinsequences from 14 different*Aspergillus*species in GenBank.** The beta-tubulin sequence from *A. flavus* A3.2890 showed the highest homology with the beta-tubulin genes from *A. flavus, A. fasciculatus*, *A. oryzae, A. subolivaceus* and *A. kambarensis.* Note that beta-tubulin genes are less effective in discriminating these closely related strains, as observed by Varga et al. (2011). Click here for file

Additional file 5 **Evaluation of peptone from different suppliers.** AF productions, as showed by TLC analyses, by *A. flavus* A3.2890 cultured in PMS (B) media made by peptone from 3 different sources for 3 days with the initial spore densities of 10^2^, 10^4^, and 10^6^ spores/ml. Three brands of peptone were purchased from Aoboxing, Sigma and Shuangxuan. Click here for file

Additional file 6 **AF contents in mycelia of*A. flavus*A3.2890 cultured in PMS and GMS media.** In PMS media, high initial spore density led to reduced AF contents in mycelia, while in GMS media high initial spore density led to increased AF contents in mycelia. The AFs were extracted from mycelia after 3-day incubation. P4 and P6: mycelia cultured in PMS media with initial spore densities of 10^4^ and 10^6^ spore/ml, respectively; G4 and G6: mycelia cultured in GMS media with initial spore densities of 10^4^ and 10^6^ spores/ml, respectively. Click here for file

Additional file 7 Primers and PCR schemes used for qRT-PCR analyses. Click here for file
