# Supplementary figures and images for: Aspergillus flavus grown in peptone as the carbon source exhibits spore density- and peptone concentration-dependent aflatoxin biosynthesis
Source: BMC Microbiol. 2012 Jun 13;12:106. doi: 10.1186/1471-2180-12-106 (PMC3412747; doi:10.1186/1471-2180-12-106)

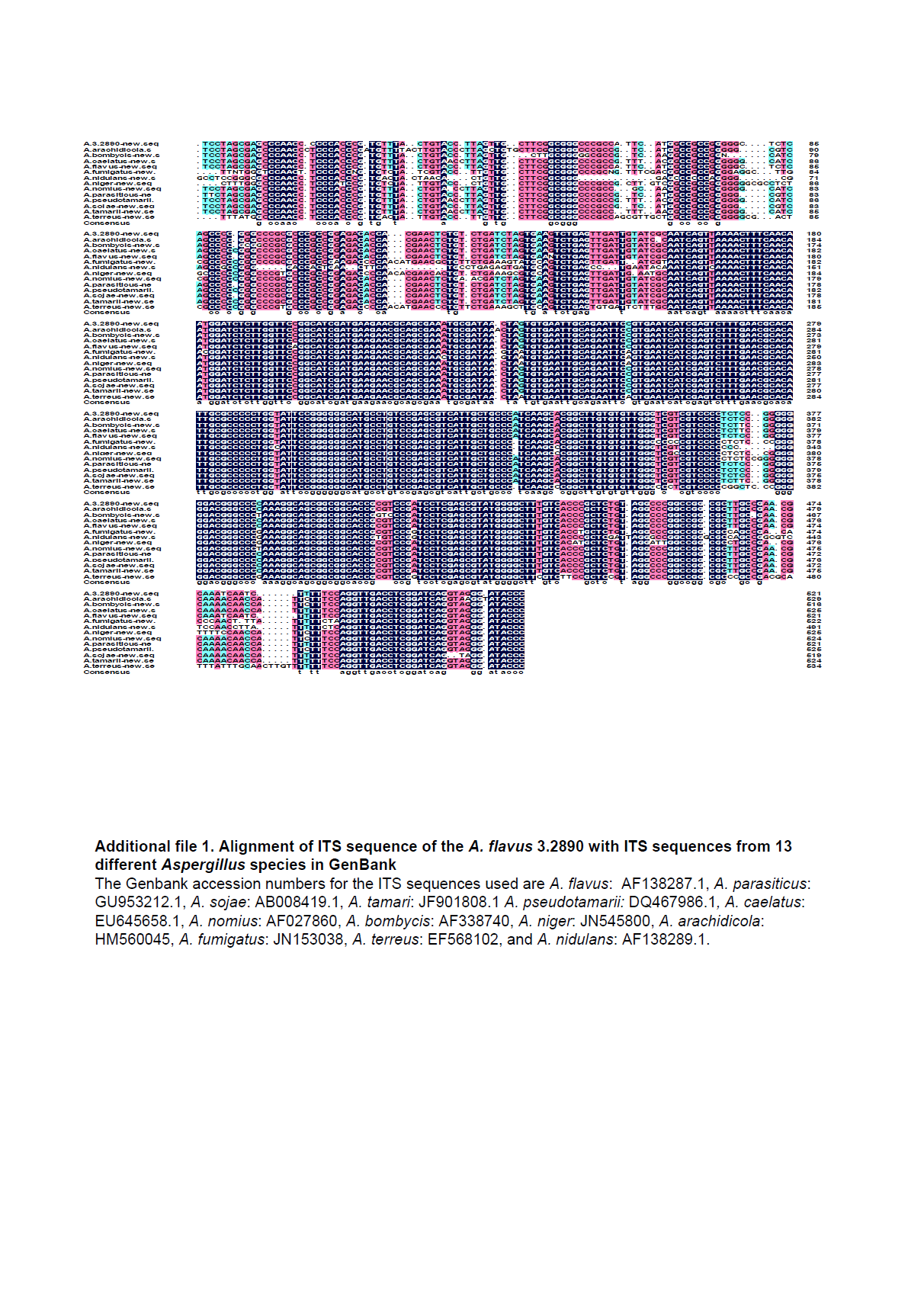

Supplement: Additional file 1 — Alignment of ITS sequence of theA. flavusA3.2890 with ITS sequences from 13 differentAspergillusspecies in GenBank. The Genbank accession numbers for ITS sequences used are A. flavus: AF138287.1, A. parasiticus: GU953212.1, A. sojae: AB008419.1, A. tamari: JF901808.1 A. pseudotamarii: DQ467986.1, A. caelatus: EU645658.1, A. nomius: AF027860, A. bombycis: AF338740, A. niger: JN545800, A. arachidicola: HM560045, A. fumigatus: JN153038, A. terreus: EF568102, and A. nidulans: AF138289.1. [file 1471-2180-12-106-S1.bmp]

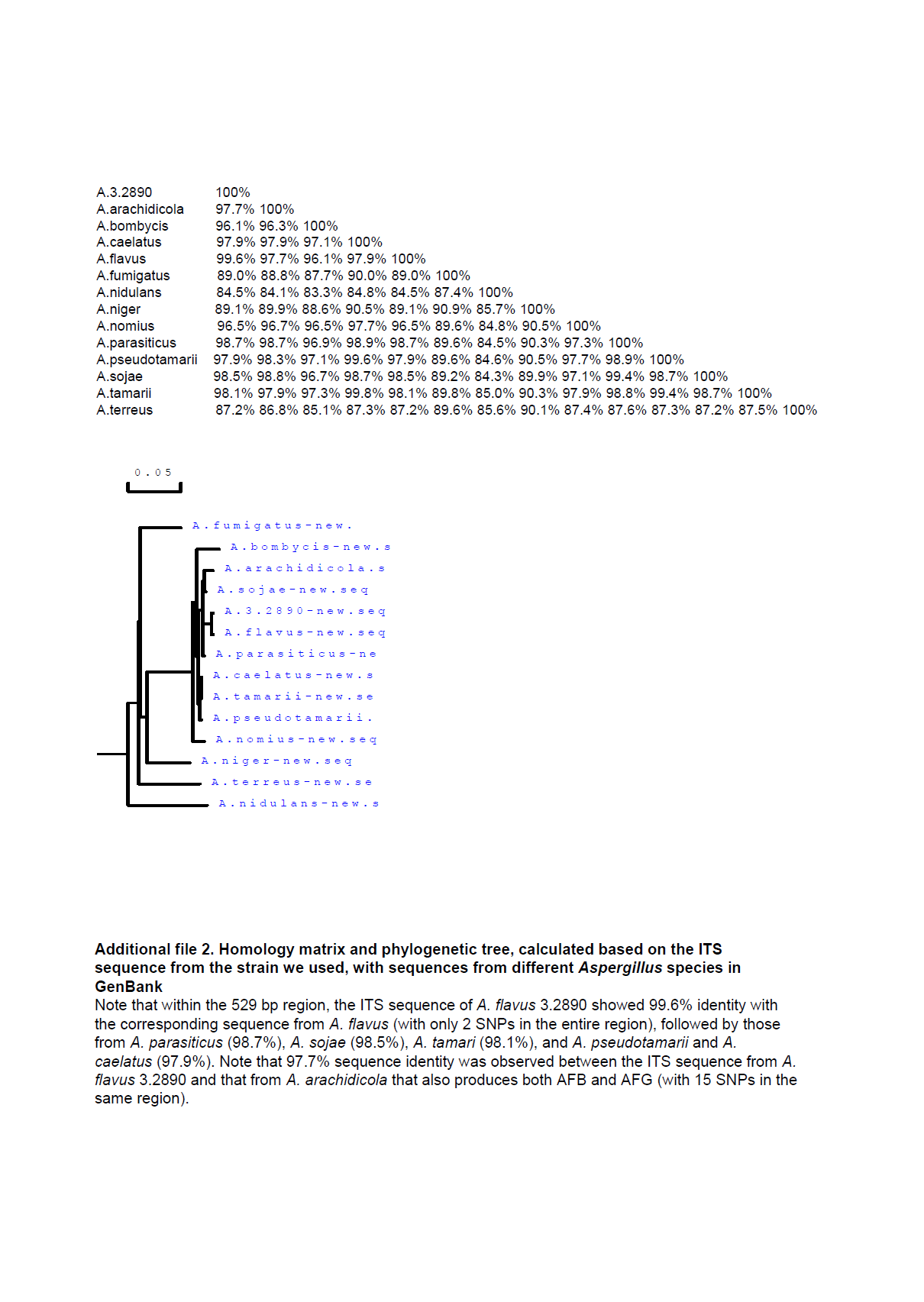

Supplement: Additional file 2 — Homology matrix and phylogenetic tree, calculated based on comparison among the ITS sequence ofA. flavusA3.2890 and sequences from differentAspergillusspecies in GenBank. Note that within the 529 bp region, the ITS sequence of A. flavus A3.2890 showed 99.6% identity with the corresponding sequence from A. flavus (with only 2 SNPs in the entire region), followed by those from A. parasiticus (98.7%), A. sojae (98.5%), A. tamari (98.1%), and A. pseudotamarii and A. caelatus (97.9%). Note that 97.7% sequence identity was observed between the ITS sequence from A. flavus A3.2890 and that from A. arachidicola that also produces both AFB and AFG (with 15 SNPs in the same region). [file 1471-2180-12-106-S2.bmp]

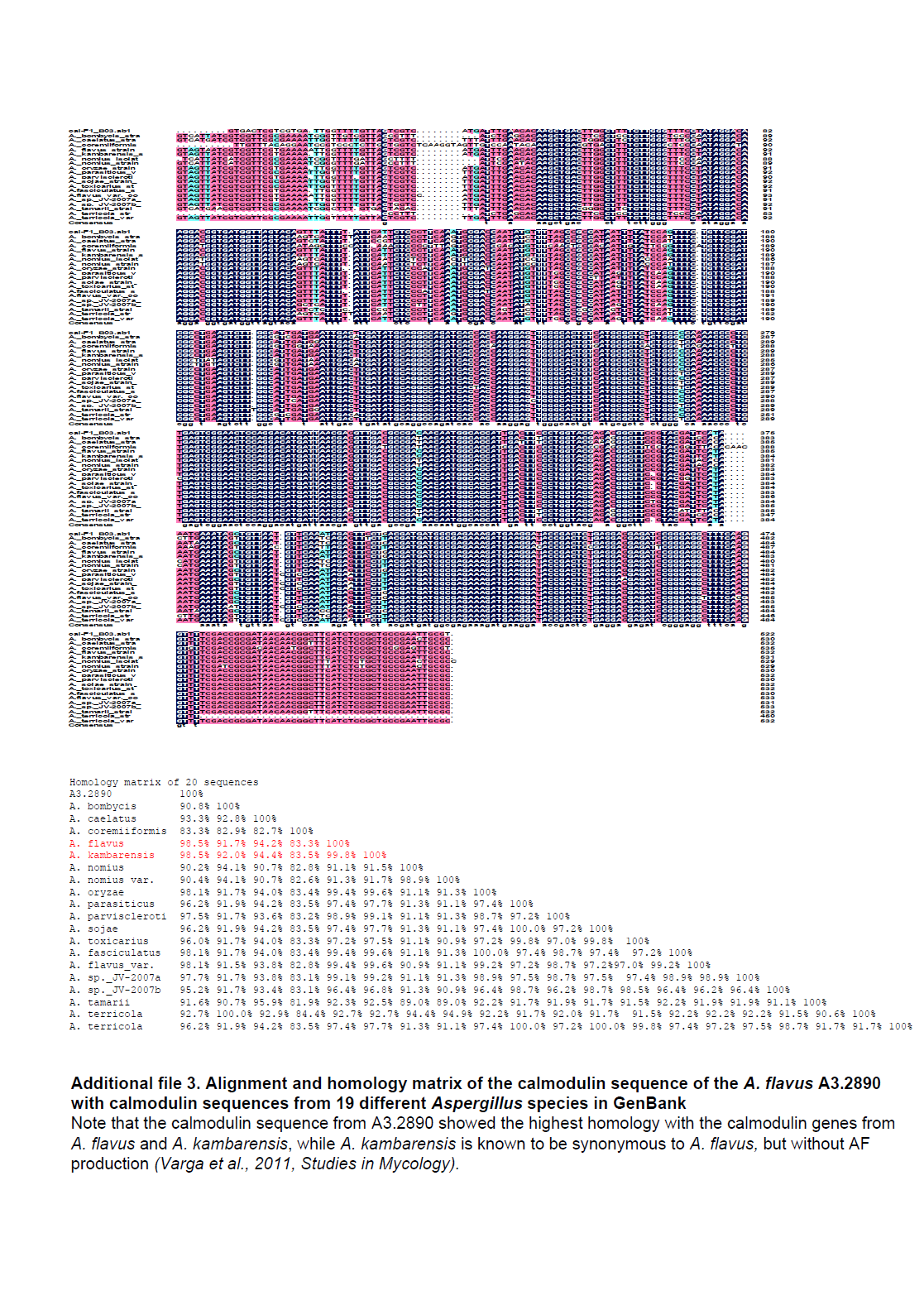

Supplement: Additional file 3 — Alignment and homology matrix of the calmodulin sequence of theA. flavusA3.2890 with calmodulin sequences from 19 differentAspergillusspecies in GenBank. Note that the calmodulin sequence from A. flavus A3.2890 showed the highest homology with the calmodulin genes from A. flavus and A. kambarensis, while A. kambarensis is known to be synonymous to A. flavus, but without AF production (Varga et al., 2011). [file 1471-2180-12-106-S3.bmp]

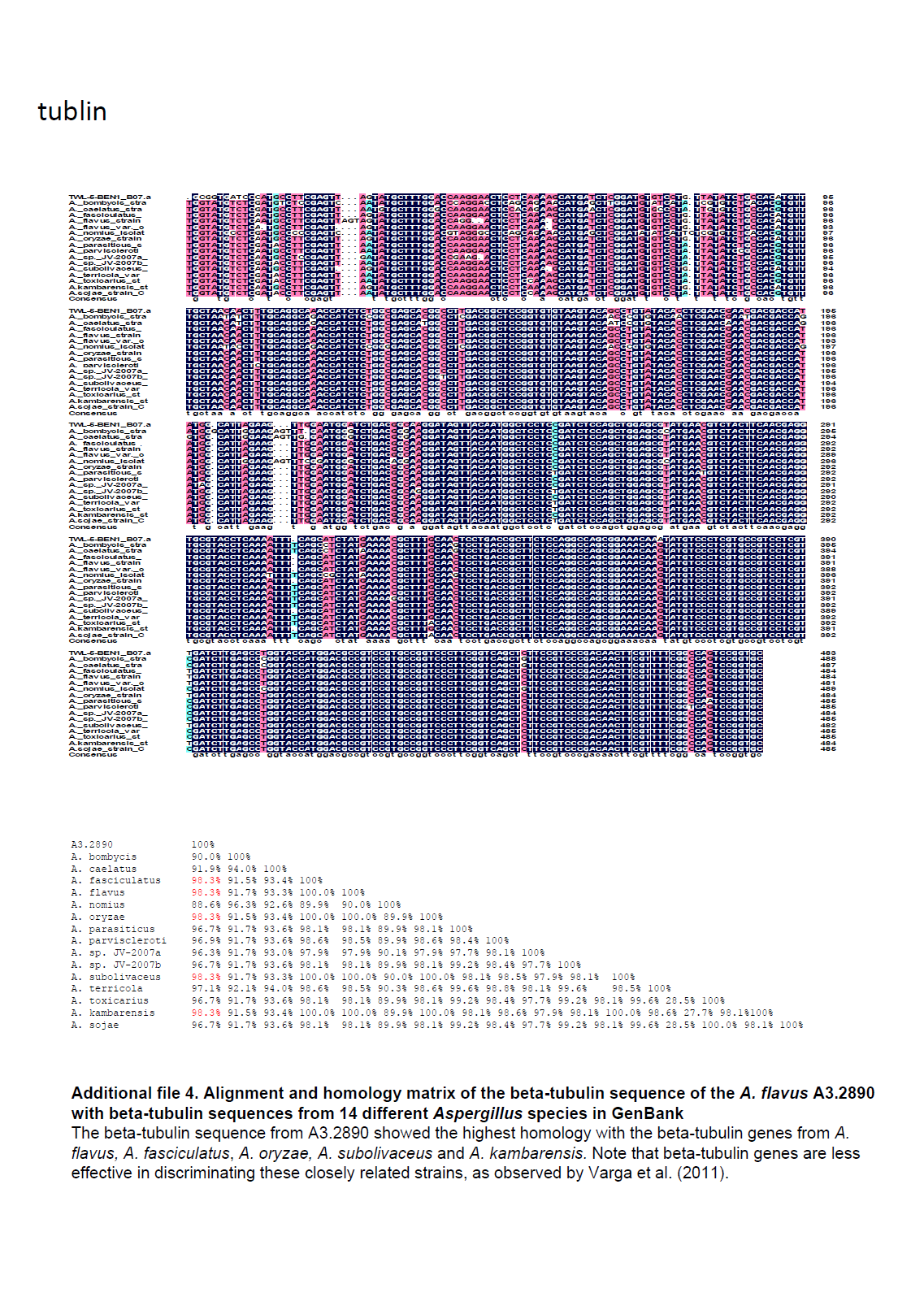

Supplement: Additional file 4 — Alignment and homology matrix of the beta-tubulin sequence of theA. flavusA3.2890 with beta-tubulinsequences from 14 differentAspergillusspecies in GenBank. The beta-tubulin sequence from A. flavus A3.2890 showed the highest homology with the beta-tubulin genes from A. flavus, A. fasciculatus, A. oryzae, A. subolivaceus and A. kambarensis. Note that beta-tubulin genes are less effective in discriminating these closely related strains, as observed by Varga et al. (2011). [file 1471-2180-12-106-S4.bmp]

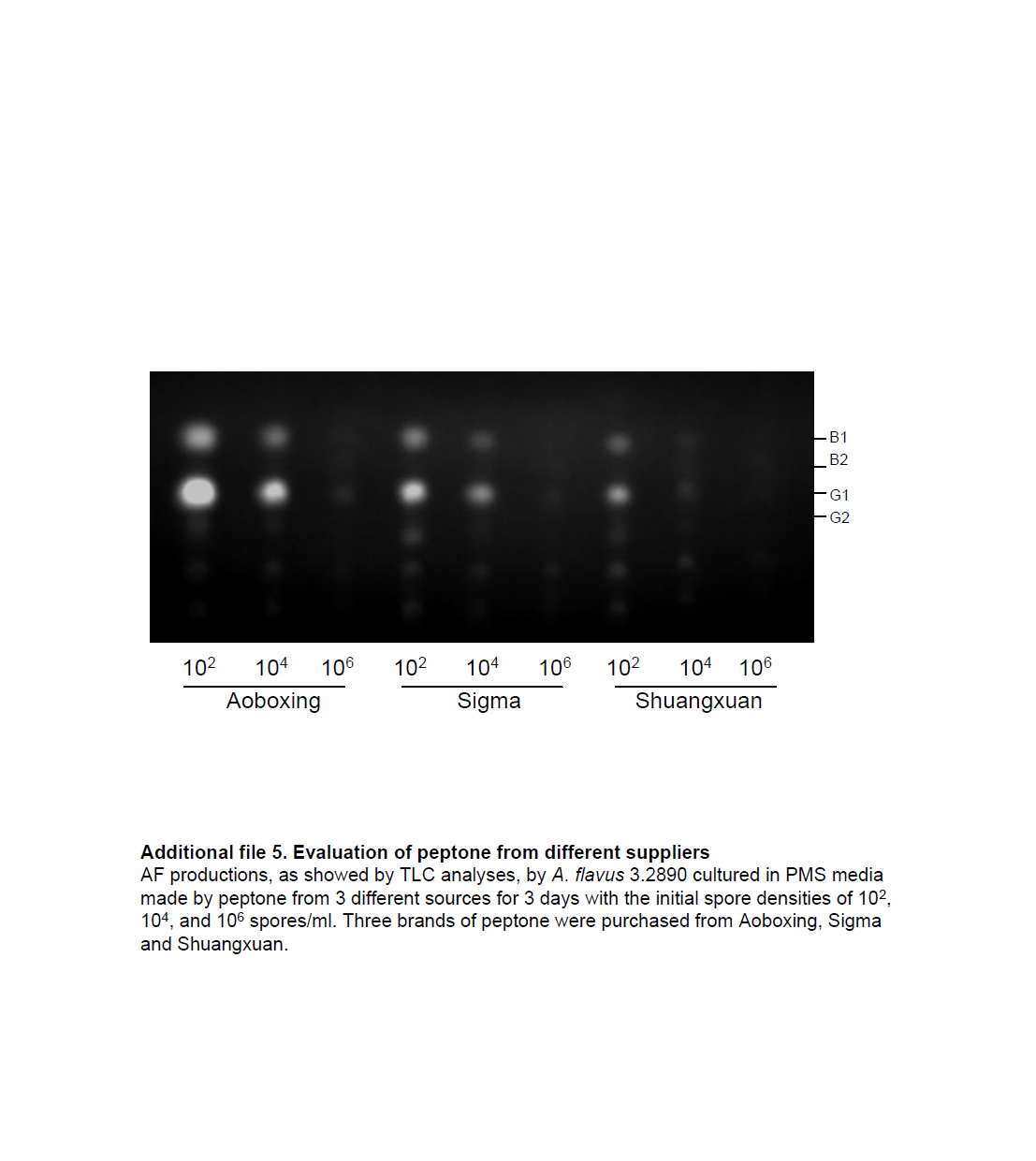

Supplement: Additional file 5 — Evaluation of peptone from different suppliers. AF productions, as showed by TLC analyses, by A. flavus A3.2890 cultured in PMS (B) media made by peptone from 3 different sources for 3 days with the initial spore densities of 102, 104, and 106 spores/ml. Three brands of peptone were purchased from Aoboxing, Sigma and Shuangxuan. [file 1471-2180-12-106-S5.bmp]

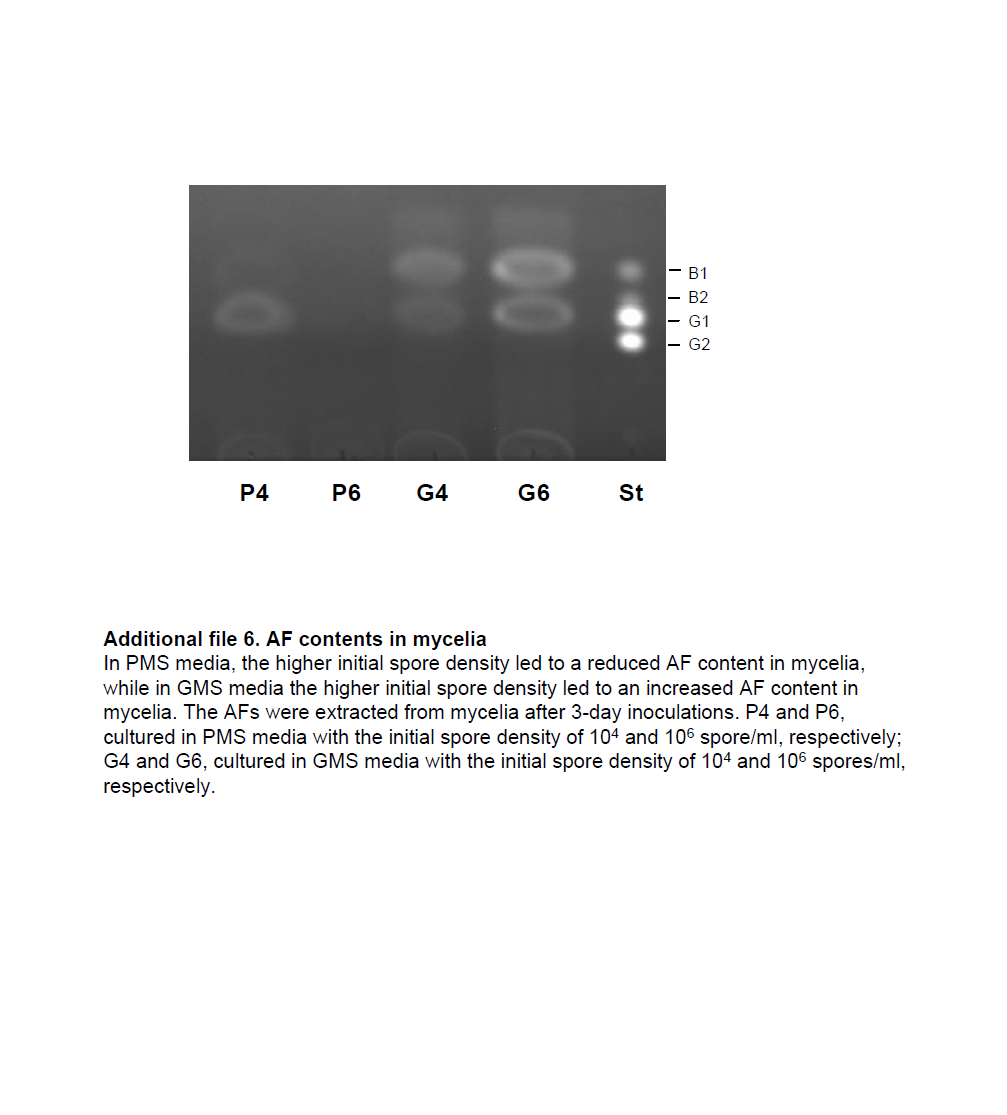

Supplement: Additional file 6 — AF contents in mycelia ofA. flavusA3.2890 cultured in PMS and GMS media. In PMS media, high initial spore density led to reduced AF contents in mycelia, while in GMS media high initial spore density led to increased AF contents in mycelia. The AFs were extracted from mycelia after 3-day incubation. P4 and P6: mycelia cultured in PMS media with initial spore densities of 104 and 106 spore/ml, respectively; G4 and G6: mycelia cultured in GMS media with initial spore densities of 104 and 106 spores/ml, respectively. [file 1471-2180-12-106-S6.bmp]

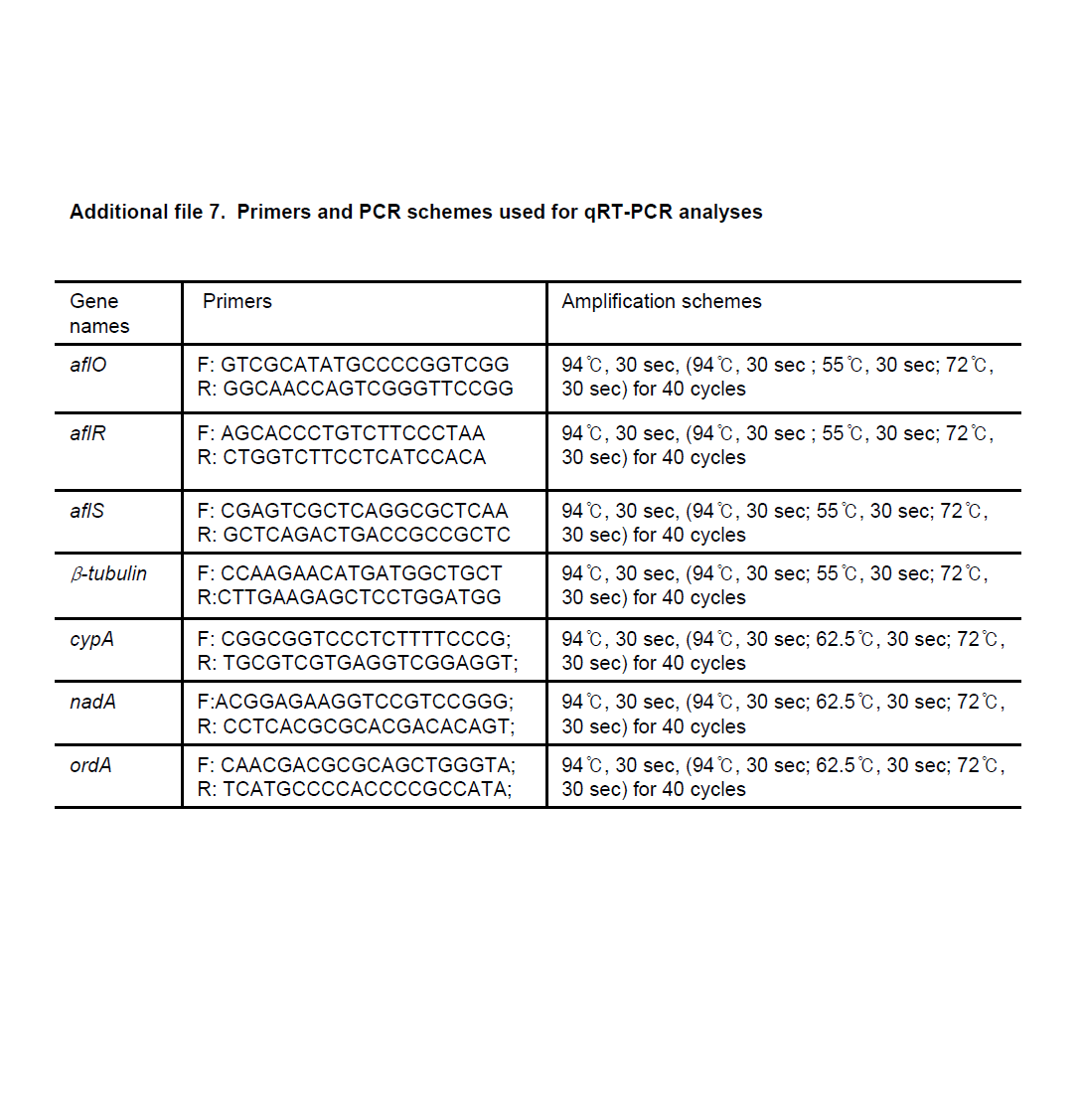

Supplement: Additional file 7 — Primers and PCR schemes used for qRT-PCR analyses. [file 1471-2180-12-106-S7.bmp]
